# Consumptıon of ultra-processed foods can accelerate age-related appearance of sarcopenıa

**DOI:** 10.1007/s10522-025-10253-8

**Published:** 2025-05-17

**Authors:** Hatice Parlak Baskurt, Hulya Yardımcı

**Affiliations:** 1 Department of Nutrition and Dietetics, Graduate School of Health Sciences, Ankara University, Ankara, Turkey; 2 Department of Nutrition and Dietetics, Faculty of Health Sciences, Kutahya Health Sciences University, Kutahya, Turkey; 3 Department of Nutrition and Dietetics, Faculty of Health Sciences, Ankara University, Ankara, Turkey

**Keywords:** Sarcopenia, Ultra-processed food, Older adults, Muscle mass, Nutrition

## Abstract

Sarcopenia, the loss of muscle mass and function, commonly affects older adults and reduces their quality of life. Frequent consumption of ultra-processed foods, which contain mostly additives and very few natural foods, increases the risk of sarcopenia or accelerates its onset. A diet rich in ultra-processed foods may lead to inadequate nutrition and lower intake of many nutrients, such as protein, dietary fiber, vitamins A, C, E, zinc, selenium, magnesium, and iron. However, obesity may occur because of increased energy and saturated fat intake. Both conditions contribute to the risk of muscle mass loss. A diet poor in antioxidants may increase the risk of sarcopenia by increasing inflammation. The fact that consumption of ultra-processed food contributes to the risk of frailty in older adults may lead to increased physical weakness and falls. Therefore, minimizing the consumption of ultra-processed foods is important to reduce the risk of sarcopenia. Understanding the contribution of the nutrients in this group to the risk of sarcopenia will allow for more accurate nutritional recommendations for old age. In our world, where the elderly population is increasing, it is important to conduct studies that include healthy nutrition to make this process healthier and more prosperous.

## Introduction

People over the age of 60 are generally referred to as older adults (United Nations Refugee Agency 2024). The number of people aged 60 and over is increasing worldwide. This number was 1 billion in 2019 and is expected to rise to 1.4 billion by 2030 and 2.1 billion by 2050 (WHO 2025). This rapid increase requires special attention to this group, which is more susceptible to diseases. Although the aging process affects everyone differently, healthy aging is a right of everyone. In this context, the years 2021-2030 have declared the "United Nations Decade of Healthy Ageing" (WHO 2024).

With aging, molecular and cellular damage accumulate in the body over time; therefore, physical and mental capacities gradually decrease. This increases the risk of diseases, such as osteoarthritis, diabetes, depression, dementia and fragility (WHO 2024), and sarcopenia may occur (Cruz-Jentoft & Sayer 2019). Although the physical times required for metabolic processes in an organism are almost identical, the transition from one biological stage to another is highly variable. The rates and extents of age-related changes were highly heterogeneous. What influences these changes is one of the knowledge gaps in modern biogerontology (Rattan 2024). Dietary regulation is one of the factors that are effective in delaying age-related changes, maintaining physical and cognitive function in old age, and preventing age-related diseases.

Sarcopenia is a progressive and common skeletal muscle disorder characterized by rapid loss of muscle mass and function. This condition is often associated with adverse outcomes such as functional decline, frailty, falls, and increased mortality (Cruz-Jentoft & Sayer 2019). Sarcopenia affects 10-16% of the elderly worldwide (Yuan & Larsson 2023). Although sarcopenia occurs with age, it is influenced by genetic and lifestyle factors across the lifespan and nutritional factors are also important among its common causes (Cruz-Jentoft & Sayer 2019).

In the NOVA (which is not an acronym) classification, which considers the industrial processes that foods undergo, foods are divided into four groups. The first group, unprocessed or minimally processed foods, includes natural foods such as fruits, milk, and eggs that can be consumed after being separated from nature or that only undergo processes such as drying, crushing, and fermentation. The second group consists of products such as oil, sugar, and salt, which are formed by processes such as refining, pressing, and grinding of unprocessed foods. The third group, processed foods, includes products such as cheese, fresh bread, and canned fish, which are usually created by combining these two groups or by increasing the taste and durability of natural foods. The fourth group consists of formulations that contain no or very few natural foods, mostly consisting of additives, and are defined as ultra-processed foods (UPF) (Monteiro et al. 2018). Examples include fast food, breakfast cereals, sodas, chocolate, nuggets, ready-made soups, energy bars, and sweet or salty packaged snacks. Ultra-processed foods generally contain additives such as salt, sugar, fat, high-fructose corn syrup, flavor enhancers, colorings, emulsifiers, sweeteners, and bulking agents (Monteiro et al. 2019). Ultra-processed foods are designed to be shelf-stable, ubiquitous, and extremely tasty and appealing (Monteiro et al. 2018). High consumption of ultra-processed foods is associated with an increased risk of obesity, cardiovascular disease, depression, cancer, and all-cause mortality (Elizabeth et al. 2020; Pagliai et al. 2021). In addition, UPFs may increase the risk of sarcopenia in old age (Almeida et al. 2022). This review discusses the relationship between sarcopenia and ultra-processed food consumption and the factors that may play a role in this relationship. Understanding this relationship may contribute to the determination of nutritional actions to prevent sarcopenia in the elderly.

### Association between ultra-processed food consumption and sarcopenia

The association between ultra-processed food consumption and sarcopenia risk, muscle strength, and mass has been investigated in several studies. A cross-sectional study compared the frequency of UPF consumption in older adults with and without sarcopenia and identified a relationship between increased UPF exposure and body composition markers of sarcopenia (Almeida et al. 2022). Sarcopenia was shown to be associated with high UPF consumption in 118 participants over the age of 60 (p = 0.004). It was found that people who consumed ultra-processed foods more than 1-2 times per week were more likely to be sarcopenic than those who consumed them less than 1-2 times per week (OR=24.85, p<0.001). The study noted that even in the presence of low exposure, UPF consumption increased the risk of developing sarcopenia in older adults. In a study examining data from 11,123 people over the age of 40 from the Korean National Health and Nutrition Examination Survey 2008–2011, UPF intake was positively associated with a higher percentage of body fat (OR_per 10%increase_, 1.04; 95% CI, 1.002-1.08) and a lower percentage of appendicular skeletal muscle mass (OR_per 10%increase_, 1.05; 95% CI, 1.01-1.09) (Jung et al. 2024). Another prospective cohort study included 5409 adults aged 40 years and older in China and showed that higher UPF consumption was associated with a greater decrease in grip strength (p < 0.01) (Zhang et al. 2022). In the adjusted models, a 10% increase in dietary UPFs was associated with a 0.3708 kg decrease in annual grip strength (95% CI, 0.5687 - 0.1730; p < 0.001).

The relationship between UPF consumption and sarcopenia has been studied in different age and disease groups. Although this topic has been addressed in the elderly, UPF consumption may also be associated with low muscle mass in the younger population. A significant linear relationship has previously been shown between higher UPF consumption and an increased risk of low muscle mass in adults aged 20-59 (n=10255). When adjusted for confounders, participants with the highest UPF intake had a 60% increased risk of low muscle mass (OR = 1.60, 95% CI: 1.13 to 2.26, P for trend = 0.003) (Kong et al. 2024). In a study of 7,173 people aged 20-59 years, higher UPF consumption was significantly associated with greater reductions in total and regional muscle mass (Sun et al. 2024). In the study, when the highest quantile of UPF consumption was compared with the lowest quantile, the total muscle mass index decreased by 0.93% and the appendicular muscle mass index decreased by 1.25%. The relationship between UPF consumption at ages 23-25 and body composition at ages 37-39 was investigated in Brazil (Rudakoff et al. 2022) and it was found that women with high UPF consumption had an increase in fat mass and a decrease in lean mass in the long term. In the adjusted analysis, a 10% increase in the percentage of UPF consumption was associated with a longitudinal increase of 0.8% in body fat percentage (β = 0.08; 95% CI: 0.04, 0.13; p = < 0.001), and a 0.7% decrease in lean mass percentage (β = −0.07; 95% CI: −0.11, −0.03; p = 0.001). A study of 490 adults and elderly people examined the effects of UPF consumption at different ages (Monteles Nascimento et al. 2023). An increase in subcutaneous body fat marker (skinfold thickness) was found with higher UPF consumption in individuals aged 20 to 35 years (ß: 0.04; CI: 0.03/0.09), and an inverse correlation was observed between UPF consumption and upper mid-arm circumference (ß: -0.02; CI: -0.03/ -0.01) and corrected arm muscle area (ß: -0.07; CI: -0.12/ -0.02), in participants between the ages of 36 and 59. In Brazilian participants aged 18-19 (n=1525), a one-unit increase in the percentage contribution of UPF to total dietary energy was associated with a 0.04 kg decrease in muscle mass and a 0.01 kg/m^2^ decrease in lean body mass (Viola et al. 2020). A cross-sectional study in Iran on 110 patients with chronic kidney disease found significantly higher odds for sarcopenia at the upper and lower median UPF intake after adjusting for confounding factors (OR = 3.59, 95% CI: 1.02–12.62, P = 0.046) (Shateri et al. 2024). Additionally, for kidney transplant recipients, higher UPF intake was correlated with lower muscle mass in participants recruited immediately after transplantation (r = −0.250, p = .037) (Costa et al. 2024a, b). The studies evaluating the relationship between ultra-processed foods and sarcopenia are presented in Table 1.

**Table 1:** Studies evaluating the relationship between ultra-processed foods and sarcopenia

Author, year	Study design	Population	Impact measure	Main findings
Almeida et al. 2022	Cross-sectional	118 people over 60 years old	Body composition was measured by Dual-energy X-ray absorptiometry (DXA) and UPF consumption was measured by the food frequency questionnaire (FFQ).	People who consume UPF more than 1-2 times per week are more likely to be sarcopenic than those who consume it less than 1-2 times per week.
Jung et al. 2024	Cross-sectional	11,123 people over 40 years old	Body composition was measured by DXA and UPF consumption by 24-h dietary recall.	Higher UPF consumption was associated with higher adiposity and lower skeletal muscle mass, especially in adults from rural areas and those with lower levels of education.
Zhang et al., 2022	Prospective cohort	5409 adults over 40 years old	Grip strength was measured using a handheld digital dynamometer and UPF consumption was measured via a FFQ.	UPF consumption has been associated with a faster decrease in grip strength.
Sun et al. 2024	Cross-sectional	7,173 people aged 20 to 59	Muscle mass measurements were assessed by DXA and UPF consumption assessed by 24-hour dietary recall.	Higher UPF consumption was significantly associated with lower muscle mass values.
Rudakoff et al. 2022	Cohort study	1.021 adults	DXA was used to assess lean mass and body fat distribution, and a food frequency questionnaire was used to assess UPF consumption.	In women, there was an association between UPF consumption and increases in body mass index, body fat percentage, and decreases in lean mass percentage.
Monteles Nascimento et al. 2023	Cross-sectional	490 adults and elderly	Anthropometric indicators such as waist-height ratio, triceps skinfold thickness, arm circumference, corrected arm muscle area, subscapula skinfold thickness and calf circumference were used, and food consumption was assessed with 24-hour dietary recall.	Greater UPF contributions to the diet were associated with increases in markers of subcutaneous fat for adults aged 20 to 35 years and decreases in markers of lean body mass for adults aged 36 to 59 years.
Kong et al. 2024	Cross-sectional	10.255 adults aged 20-59	UPF consumption was assessed using a 24-hour dietary recall and appendicular lean mass was assessed using DXA.	Significant linear relationships were observed between UPF consumption and low muscle mass.
Viola et al. 2020	Cross-sectional	1525 participants (aged 18-19)	Body composition was measured by air displacement plethysmography with BOD POD Gold Standard and DXA. UPF consumption was measured by FFQ.	Increased UPF consumption in the diet was associated with decreased muscle mass.
Shateri et al. 2024	Cross-sectional	110 patients with chronic kidney disease	The Asian Sarcopenia Study Group Guidelines were used for sarcopenia diagnosis (including dynamometry for handgrip strength, bioelectrical impedance analysis (BIA) to measure muscle and fat mass, and 5-times chair stand test and gait speed test to assess muscle function), while the semi-quantitative FFQ was used to assess dietary intake.	A positive association between UPF consumption and sarcopenia has been demonstrated.
Costa et al. 2024a, b	Cross-sectional	96 kidney transplant recipients	Food intake was assessed with FFQ and body composition was assessed with BIA.	For the group immediately after transplantation, UPF intake was correlated with lower muscle mass.

### Factors affecting the relationship between ultra-processed food consumption and sarcopenia

Nutritional factors that may cause sarcopenia include anorexia, malabsorption, low protein intake, and micronutrient deficiencies that develop with age (Cruz-Jentoft & Sayer 2019). The fact that UPFs are deficient in important nutrients (protein, vitamins, and minerals) for metabolic processes and have a high energy density contributes to possible negative situations. Excessive consumption of ultra-processed foods paves the way for sarcopenia by increasing deficiencies in protein, dietary fiber, vitamins A, C, and E, and minerals such as magnesium, zinc, iron, and selenium (Louzada et al. 2015a); reducing diet quality (Salomé et al. 2021); increasing the risk of frailty (Hao et al. 2022); increasing inflammation (Contreras-Rodriguez et al. 2023) and obesity (Harb et al. 2023).

### Malnutrition

Loss of appetite is common in older individuals (Cox et al. 2020)**.** Poverty, loneliness, and social isolation, along with depression and loss of appetite, can reduce food consumption among older people. Additionally, faster satiety signals, poor dental structure, and age-related changes in taste and smell can limit the type and amount of food consumed. This can lead to malnutrition in terms of protein, energy, and micronutrients (Donini et al. 2003). This condition, also called anorexia of aging, is associated with adverse outcomes, such as reduced dietary diversity, sarcopenia, and weakness (Cox et al. 2020).

An unhealthy diet exacerbates malnutrition, which can occur during the natural course of aging. As ultra-processed foods become more prevalent in the diet, the intake of protein and some vitamins and minerals decreases (Costa et al. 2024a, b). It has been reported that the consumption of high amounts of such foods is inversely proportional to the dietary content of vitamins B_12_, D, E, niacin, pyridoxine, copper, iron, phosphorus, magnesium, selenium, and zinc (Louzada et al. 2015a). Increasing the UPF contribution to total energy intake in adults decreased protein, fiber, copper, and vitamin E intake (Costa et al. 2024a, b). The proportion of UPF in the diet was inversely correlated with the content of protein, fiber, vitamins A, C, D, B6, B12, niacin, thiamine, riboflavin, zinc, iron, magnesium, calcium, phosphorus and potassium (Moubarac et al. 2017). In contrast to ultra-processed foods, a higher consumption of unprocessed foods is associated with higher animal protein intake and better plant protein diversity (Salomé et al. 2021). These results suggest that increased consumption of UPF may contribute to malnutrition by reducing the intake of quality protein, vitamins, and minerals.

A diet deficient in protein and energy is associated with impaired muscle function (Donini et al. 2003). According to a meta-analysis, the average amount of energy and nutrients consumed was significantly lower in sarcopenic elderly than in non-sarcopenic elderly; people with sarcopenia had a lower intake of protein, carbohydrates, saturated fatty acids, vitamins A, B_12_, C and D; and minerals such as calcium, magnesium, sodium and selenium (Santiago et al. 2021). However, one study found no significant association between appetite loss and frailty in older adults (Zukeran et al. 2022)**.**

### Low diet quality

A high-quality and healthy diet throughout adulthood is linked to the effective maintenance of muscle mass and function (Robinson et al. 2023). However, UPFs are associated with unhealthy dietary profiles because they are rich in energy, starch, sugar, salt, saturated fats, and trans fats, and poor in protein, dietary fiber, some micronutrients, and other bioactive compounds. (Monteiro et al. 2018).

In a study on French adults, diet quality scores were negatively associated with UPF (Salomé et al. 2021). The diet quality of adults in Korea was assessed using the Korean Healthy Eating Index, and the proportion of UPF in daily energy intake was inversely correlated with the diet quality score and was associated with poor diet quality (Shim et al. 2022). Similarly, the percentage of energy from the UPF was inversely related to the diet quality in Australian adults. In the study, diet quality was examined using the Dietary Guidelines Index, and a higher percentage of energy from UPF was associated with lower Dietary Guidelines Index scores for dietary diversity, consumption of fruits, vegetables, and grains, and limitation of saturated fat and extra sugar (Marchese et al. 2022). Nutritional frailty was defined as the coexistence of physical frailty and nutritional imbalance in 2185 older adults who underwent nutritional assessment, 27% of whom had nutritional frailty. With the increased consumption of ultra-processed foods, the likelihood of nutritional frailty has also increased. The contribution of processed foods to malnutrition during aging has been emphasized (Zupo et al. 2023). In conclusion, the association between UPFs and poor diet quality has been demonstrated in various studies. However, in older adults, healthy diets can reduce the risk of decline in physical performance (Bloom et al. 2018), and diet quality defined by variety and nutrient adequacy can be positively associated with sarcopenia components such as muscle mass, muscle strength and physical performance (Ramadas et al. 2022). Therefore, it can be considered that healthy and high-quality diets low in UPF would be beneficial in preventing sarcopenia.

### The role of frailty

In the elderly, function in various organ systems decreases with age and frailty, a multifactorial syndrome that occurs with a decrease in resistance to stress factors (Cesari et al. 2017). Frailty includes decreased physical strength and sarcopenia (Cruz-Jentoft et al. 2017; Cruz-Jentoft & Sayer 2019). The risk of falls and need for medical care are increased in frail older adults (Cai et al. 2020). UPF is likely to increase frailty in old age, and increased UPF consumption in younger populations may pose a risk of compromising healthy aging in the long term (Mariath et al. 2022).

In a study analyzing data from 2,329 elderly participants in the United States, energy and energy ratios from ultra-processed foods were found to be positively associated with the risk of frailty in underweight, normal-weight and overweight individuals (Hao et al. 2022). In a prospective cohort study of 1,822 individuals aged ≥ 60 years recruited in Spain between 2008 and 2010, with a mean follow-up of 3.5 years, UPF consumption was strongly associated with the risk of frailty (Sandoval-Insausti et al. 2020). A study that followed 63,743 women aged 60 years and older for 26 years found that UPFs were directly associated with frailty risk (Fung et al. 2024). Frailty in older adults is associated with increased UPF consumption (Clayton-Chubb et al. 2024). Healthier dietary profiles, such as increased consumption of fruits and vegetables and reduced consumption of UPF, are associated with a lower risk of frailty (Ni Lochlainn et al. 2021).

### Obesity

Both malnutrition and obesity play an important role in the pathogenesis of frailty and sarcopenia (Cruz-Jentoft et al. 2017). Higher consumption of ultra-processed foods is associated with overweight and obesity (Harb et al. 2023; Poti et al. 2017).

As the proportion of UPF in total energy intake increases, there is a trend towards increased intake of energy, fats, carbohydrates, calcium and manganese, thiamine and vitamin B_6_ (Costa et al. 2024a, b). Consumption of ultra-processed foods has higher energy density, total fat content, saturated and trans fatty acids, and free sugars compared to natural foods (Louzada et al. 2015b). A systematic review and meta-analysis of observational studies found a positive association between UPF and excess body weight (Askari et al. 2020). The Brazilian study found that individuals in the top quartile of household consumption of ultra-processed products were 37% more likely to be obese than those in the bottom quartile (Canella et al. 2014).

According to a systematic review, the highest UPF consumption was associated with abdominal obesity in prospective cohort studies of adults over 60 years of age. In cross-sectional studies, the highest UPF consumption was associated with an unhealthy diet (Shahatah et al. 2025).

### Inflammation

One of the pathways to sarcopenia is mitochondrial dysfunction that develops with aging and the resulting oxidative stress and inflammation (Romani et al. 2022). Oxidative stress with aging is caused by both a decrease in the availability of dietary antioxidants and the accumulation of products derived from the oxidation of biological structures (Junqueira et al. 2004). Damaged and dysfunctional mitochondria, which accumulate in sarcopenic muscles with age-related decrease in mitochondrial content and dysfunction, are the main source of cellular reactive oxygen species (ROS) (Romani et al. 2022). Although ROS are formed as natural products of normal cellular activity, their overproduction can cause oxidative stress (Shlapakova et al. 2020). In addition, ROS are involved in the initiation, progression and resolution of the inflammatory response (Chelombitko 2018). Antioxidant defense minimizes ROS levels to prevent oxidative damage (Shlapakova et al. 2020).

Inflammation is a defense mechanism caused by tissue damage or metabolic stress and restores homeostasis in the damaged area. This process ends when the threat is eliminated, but inflammation can become chronic when it does not work properly or when the threat persists (Calder et al. 2013). Overproduction of ROS can contribute to cell and tissue damage and chronic inflammation (Chelombitko 2018). Aging is associated with oxidative stress, various changes in cellular structures and macromolecules, and DNA damage (Shlapakova et al. 2020).

Although inconsistent evidence has been reported on the association between the consumption of ultra-processed foods and inflammation (Tristan Asensi et al. 2023), excessive consumption of these foods may increase some inflammatory markers and lead to a more inflammatory profile in individuals (Contreras-Rodriguez et al. 2023; Quetglas-Llabrés et al. 2023). This may be due to malnutrition, as excess consumption of UPF results in a lower intake of various nutrients (Moubarac et al. 2017). The risk of inflammation is increased in the case of malnutrition, when adequate and nutrient-dense food is not consumed (Parlak Baskurt & Yardımcı, 2024). Another reason may be that a diet rich in UPFs contains less antioxidant elements and is rich in pro-oxidant elements. Because the effects of inadequate intake of vitamins D, E and C in increasing antioxidant activity are insufficient due to higher consumption of UPFs (Shaik-Dasthagirisaheb et al. 2013). Minerals such as selenium and zinc, whose intake can be reduced with UPF, are components of enzymes such as glutathione peroxidase and superoxide dismutase, which have antioxidant functions (Burk 2002; Prasad et al. 2004). In addition, metabolic processes such as the prebiotic properties of dietary fiber, which promote intestinal barrier function and anti-inflammatory responses, cannot occur when UPF consumption is high (Calder et al. 2009). On the other hand, polyphenols, which are abundant in unprocessed natural foods such as fruits and vegetables, exert anti-inflammatory effects. They do this by activating the gene transcription factor PPAR-γ, which inhibits the activation of nuclear factor kappaB, which stimulates the synthesis of inflammatory products (Sears 2015). High UPF consumption together with high simple sugar and saturated fat intake may lead to changes in gut microbiota and inflammatory responses. Overly processed diets have been associated with biochemical changes such as oxidative stress, inflammation and gut dysbiosis (Martínez Leo et al. 2021).

## Conclusion

Excessive consumption of ultra-processed foods in older adults increases the risk of sarcopenia by affecting many aspects. Future longitudinal studies may be useful in understanding the relationship between UPF consumption and muscle mass and function. A healthy and balanced diet with low UPF in the elderly population reduces or delays the risks of sarcopenia and improves quality of life. 

## Data Availability

No datasets were generated or analysed during the current study.
